# Formation of a vertical SnSe/SnSe_2_ p–n heterojunction by NH_3_ plasma-induced phase transformation

**DOI:** 10.1039/d2na00434h

**Published:** 2022-11-25

**Authors:** Yi Li, Juanmei Duan, Yonder Berencén, René Hübner, Hsu-Sheng Tsai, Chia-Nung Kuo, Chin Shan Lue, Manfred Helm, Shengqiang Zhou, Slawomir Prucnal

**Affiliations:** a Helmholtz-Zentrum Dresden-Rossendorf, Institute of Ion Beam Physics and Materials Research Bautzner Landstrasse 400 D-01328 Dresden Germany y.li@hzdr.de s.prucnal@hzdr.de; b Technische Universität Dresden D-01062 Dresden Germany; c Department of Physics, National Cheng Kung University Tainan 70101 Taiwan; d Taiwan Consortium of Emergent Crystalline Materials, Ministry of Science and Technology Taipei 10601 Taiwan

## Abstract

Layered van der Waals crystals exhibit unique properties making them attractive for applications in nanoelectronics, optoelectronics, and sensing. The integration of two-dimensional materials with complementary metal-oxide-semiconductor (CMOS) technology requires controllable n- and p-type doping. In this work, we demonstrate the fabrication of vertical p–n heterojunctions made of p-type tin monoselenide (SnSe) and n-type tin diselenide (SnSe_2_). The p–n heterojunction is created in a single flake by the NH_3_-plasma-assisted phase transformation from SnSe_2_ to SnSe. We show that the transformation rate and crystal quality strongly depend on plasma parameters like plasma power, temperature, partial pressure, NH_3_ flow, and duration of plasma treatment. With optimal plasma parameters, the full transformation of SnSe_2_ flakes into SnSe is achieved within a few seconds. The crystal quality and the topography of the fabricated SnSe–SnSe_2_ heterostructures are investigated using micro-Raman spectroscopy and cross-sectional transmission electron microscopy. The formation of a p–n junction is verified by current–voltage measurements.

## Abstract 2

Layered van der Waals crystals exhibit unique properties making them attractive for applications in nanoelectronics, optoelectronics, and sensing.

## Graphical abstract

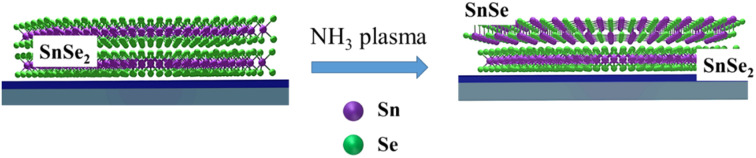

## Introduction

Graphene is the most investigated 2D material, but due to the lack of a bandgap, the potential application of graphene is rather limited. Nevertheless, the discovery of ultra-high carrier mobility in atomically thin graphene layers and outstanding optical properties^1^ stimulated scientists to explore other materials, namely layered van der Waals crystals.^2–7^ In contrast to graphene, most of them have an optical bandgap in the energy range from about 6 eV for h-BN down to tens of meV for different transition metal dichalcogenides (TMDs) or graphene ribbons.^8^ The as-synthetized mono- and dichalcogenides have well-defined native conductivity, which can be changed by external doping or by defect engineering. Moreover, the mono- or a few-layer-thick 2D mono- and dichalcogenides possess a direct bandgap with an emission in the visible and mid-infrared spectral range, making them attractive for optoelectronics.

SnSe is a p-type semiconductor with an orthorhombic crystal structure.^9^ The indirect and direct bandgaps of SnSe are 0.9 and 1.2 eV, respectively. SnSe is thermally stable with a melting point as high as 880 °C and can be kept in an ambient environment for a few weeks without detectable degradation. It can be fabricated by selenization of Sn thin films at about 550–570 °C.^10^ Fernandes *et al.* showed that selenization of Sn thin films in the temperature range of about 300 to 470 °C leads to the formation of SnSe_2_ with a trigonal CdI_2_-type crystal structure of C_6_ space symmetry.^11^ The as-synthetized SnSe_2_ is contaminated with Se atoms at the surface which can be later removed by annealing in a vacuum. In contrast to monoselenide, SnSe_2_ is an n-type semiconductor with an indirect bandgap of about 0.95 eV, and the allowed direct optical transition is at about 2.5 eV, while the band gap of bulk SnS_2_ is 2.18 eV and that of monolayer SnS_2_ is 2.41 eV, respectively. It was shown that both SnSe and SnSe_2_ are attractive for photovoltaics due to their efficient light harvesting ability, and they show outstanding thermoelectric properties with a figure of merit *ZT* factor as high as 2.2.^12^ Combining SnSe_2_ with SnSe allows the fabrication of vertical p–n junctions. Besides selenization of Sn, mono- and dichalcogenides can be synthetized using a wide variety of fabrication techniques, like solvothermal and chemical bath deposition,^13–15^ electrodeposition, spray pyrolysis,^16,17^ chemical vapor deposition or pulsed laser deposition.^11^ To this day, the fabrication of either lateral or vertical heterostructures on the same substrate is challenging due to the different growth conditions for different materials. The phenomenon of phase transformation observed on some of the chalcogenides is a very promising method to fabricate vertical p–n junctions. The phase transformation in SnSe_2_ and SnSe bulk crystals was reported by Albers and Verberkt for the first time.^18^ Single-crystalline SnSe and SnSe_2_ can be grown by saturation of the Sn–Se melt with Se or Sn, respectively. SnSe_2_–SnSe mixed crystals were obtained by changing the solidification speed. Sutter *et al.* showed that using high-energy electrons, both SnSe_2_ and SnS_2_ can be converted into monoselenide and monosulfide, respectively.^19^ According to DFT calculations, the electron energy required to promote the phase transformation of dichalcogenides into monochalcogenides must be in the range of 80 keV or higher.^19^ Due to the deep penetration depth of highly energetic electrons into Sn-dichalcogenides, monochalcogenides are created simultaneously at different depths and finally, the whole flake/layer is converted into the corresponding monochalcogenide. Zhou *et al.* used high-temperature annealing of SnS_2_ flakes in an Ar atmosphere for phase transformation.^20^ In particular, they showed that after annealing at 600 °C in a continuous Ar flow, SnS_2_ flakes were converted into monosulfides due to the sublimation of S atoms from the surface. A similar effect was obtained by annealing Sn-dichalcogenides in a vacuum. The full conversion of the dichalcogenides into monochalcogenides occurred after annealing at about 500 °C for 90 min.^21^ More precise control of the transformation of dichalcogenides into monochalcogenides is achieved by applying an Ar plasma. Recently, Kim *et al.* used an Ar plasma to remove S atoms from the surface of SnS_2_ films, and the formation of p–n vertical junctions between SnS and SnS_2_ was demonstrated.^22^

In this work, we present the fabrication of vertical p–n heterojunctions using NH_3_-plasma-assisted phase transformation of n-type trigonal dichalcogenides (SnSe_2_) into p-type orthorhombic monochalcogenides (SnSe). Cross-sectional transmission electron microscopy (TEM) images reveal an atomically flat interface between SnSe_2_ and SnSe. We also show that the phase change and crystal quality of the transformed flakes strongly depend on plasma power, time, partial pressures, and temperature. The formation of the p–n heterojunction is confirmed by the rectifying behavior of the current–voltage characteristics and the change of the phonon spectra. After NH_3_ plasma treatment, the investigated flakes exhibit four new Raman peaks which are identified as the A_g_^1^ (70 cm^−1^), B_3g_ (100 cm^−1^), A_g_^2^ (120 cm^−1^), and A_g_^3^ (147 cm^−1^) Raman-active phonon modes in SnSe.^23^

## Experimental part

SnSe and SnSe_2_ bulk crystals were synthetized by the Bridgman method. For the SnSe crystal, a stoichiometric ratio between Sn and Se (99.999% purity for each element) was used. Sn and Se were loaded into a small quartz tube and sealed into a second ampoule to avoid cracking upon cooling. The charged ampoule was heated in a vertical furnace to 910 °C for 12 h and kept at 910 °C for 24–48 h for homogenization. After that, the ampoule was slowly cooled to 750 °C at a rate of 1 °C h^−1^ and kept at this temperature for 50 h. A similar process was used to fabricate SnSe_2_. Due to the lower melting point, the dwell temperature was reduced to 700 °C. After the successful fabrication of bulk crystals, micro-flakes with layer thicknesses from a mono-layer to tens of nanometers were obtained by conventional mechanical exfoliation using Nitto tape SPV 224PR-MJ. SnSe and SnSe_2_ flakes were transferred onto a Si wafer covered with 75 nm of SiN_*x*_. The SiN_*x*_ layer was deposited on the Si wafer using plasma-enhanced chemical vapor deposition (PECVD) at 350 °C. The SiN_*x*_ films show better optical contrast for thin 2D materials than the conventionally used 290 nm thick SiO_2_.^24^ To improve the adhesion between the flakes and the substrates, the samples with the tape (just before transfer) were heated up to 70 °C for 4 min and the tape was taken out. We inspected the influence of different N_2_ and NH_3_ plasma powers, times, temperatures, and partial pressures on the phase-change phenomenon in SnSe_2_. For the sake of comparison, the SnSe flakes were processed under the same conditions. Shortly after exfoliation, the samples with SnSe_2_ were placed in a PECVD load-lock chamber. Our PECVD system allows for both the deposition of a SiN_*x*_ capping layer and the N_2_/NH_3_ plasma treatment. Reference SnSe and SnSe_2_ samples were covered with a 6 nm-thick SiN_*x*_ film deposited by PECVD at 200 °C, the temperature at which no phase change was detected. The growth rate of SiN is about 10 nm min^−1^. The SiN_*x*_ layer is used to protect the 2D materials from contamination. The N_2_ or NH_3_ plasma treatment was performed prior to SiN_*x*_ capping layer deposition without breaking the vacuum. The NH_3_ plasma parameters are summarized in Table 1. The same parameters were used for N_2_ plasma treatment, but no phase transformation was detected. Here, we present the data obtained for a plasma power of 20 W.

**Table 1:** NH_3_ plasma parameters (temperature, time, pressure, and plasma power) tested for the phase transformation of SnSe_2_ and optimal parameters for the formation of an SnSe_2_/SnSe phase mixture based on micro-Raman analyses. Three temperatures (250, 300 and 350 °C) are the optimal NH_3_ plasma temperatures for the formation of an SnSe_2_/SnSe heterojunction

Temperature (°C)	Time (s)	Pressure (mTorr)	Plasma power (W)	SnSe_2_ phase	SnSe phase	Phase Mixture
200	<60	500–800	20	√	—	—
250	5–30	500–800	20			√
300	5–30	500–800	20			√
350	5–30	500–800	20			√
400	5–30	500–800	20		√	

Increasing the plasma power considerably above 20 W makes it difficult to control the phase transformation, while lower power reduces the concentration of H ions in the plasma, making the process less efficient. If the pressure drops significantly below 500 mTorr, the plasma treatment time must increase clearly beyond one minute. For a too-high pressure, on the other hand, the phase transformation cannot be controlled and some etching of the sample surface is even visible. The same is observed for samples annealed at 400 °C or a higher temperature, where only SnSe is recorded. Additionally, the plasma treatment time strongly influences the phase change of SnSe_2_. Below 5 s, no change was observed, while above 30 s, only SnSe was obtained.

**Fig. 1:**
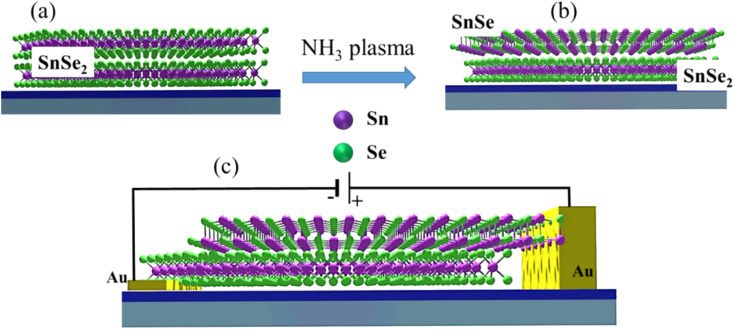
Schematic representation of (a and b) the phase transformation of SnSe_2_ into SnSe subjected to NH_3_-plasma treatment and (c) the schematics of the electrical connections to the SnSe/SnSe_2_ heterojunction.

Micro-Raman spectroscopy was performed using a 532 nm laser for excitation and a liquid-nitrogen-cooled Si-CCD to record the signal. All spectra were measured in the wavenumber range between 50 and 550 cm^−1^. To investigate the microstructural properties of the heterostructures, in particular, the phase transformation under NH_3_ plasma treatment, cross-sectional high-resolution transmission electron microscopy (HR-TEM) experiments were performed on a Titan 80–300 microscope (FEI). Moreover, spectrum imaging analysis based on energy-dispersive X-ray spectroscopy (EDX) was used to determine the element distribution after the plasma treatment using a Talos F200X microscope (FEI) operated in scanning TEM mode. For the electrical measurements, pre-patterned substrates with Au contacts were used. First, 100 nm-thick gold markers and contacts were prepared with optical lithography and a lift-off method. Next, the SnSe_2_ flakes were transferred onto the top of the gold contacts so that either the flake was placed on top of two contacts or only one side of the flake was lying on the Au stripe. After NH_3_-plasma treatment, the part of the SnSe-converted flake outside the gold contact was electrically connected with a tungsten needle of about 700 nm diameter (Fig. 1). The electrical properties of the SnSe_2_ flake and the SnSe_2_–SnSe p–n junction were measured with the help of a probe station equipped with a microscope and a Keithley 6400 power supply.

## Results and discussion

**Fig. 2:**
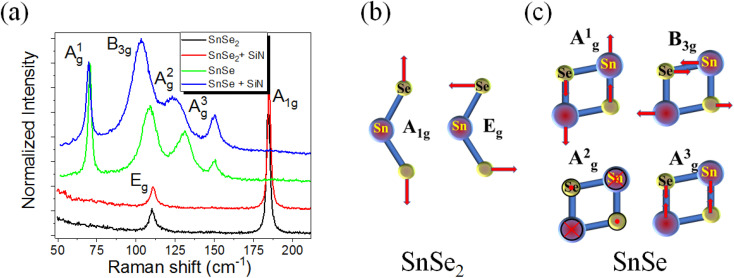
(a) Micro-Raman spectra of the reference sample obtained from the as-exfoliated SnSe_2_ and SnSe flakes transferred onto an SiN_*x*_/Si substrate and capped with a 6 nm-thick SiN_*x*_ film. All spectra were intentionally shifted along the vertical axis for clarity. Panels (b) and (c) show the atomic displacement corresponding to all Raman-active phonon modes in SnSe_2_ and SnSe, respectively.

According to Raman spectroscopy, the SiN_*x*_ capping layer has almost no influence on the phonon spectrum of SnSe and SnSe_2_ thin films. The same behavior was observed for a-few-monolayer-thick MoSe_2_, indicating that a SiN_*x*_ layer can be used to protect the 2D materials from contaminations during device fabrication.^25^Fig. 2a shows the micro-Raman spectra obtained from exfoliated SnSe and SnSe_2_ layers with and without a capping layer. According to theoretical calculations, monochalcogenides are supposed to have 12 Raman-active modes for low-symmetry SnSe (4A_g_, 2B_1g_, 4B_2g_ and 2B_3g_), but only four are visible in the micro-Raman spectra in the wavenumber range of 50–200 cm^−1^; *i.e.* A_g_^1^ at 70 cm^−1^, B_3g_ at 106 cm^−1^, A_g_^2^ at 127 cm^−1^ and A_g_^3^ at 147 cm^−1^.^26^Fig. 2c shows a schematic representation of the in-plane and out-of-plane active Raman vibrational modes in SnSe. To protect the SnSe flakes from contamination, we deposited a 6 nm-thick SiN_*x*_ capping layer at 200 °C using PECVD. According to our experience, 2D flakes covered with a SiN_*x*_ layer are stable for at least two years. Based on the Raman measurements, the SiN_*x*_ capping layer does not affect the out-of-plane vibrational modes (A_g_^1^ and A_g_^3^), but the in-plane modes B_3g_ and A_g_^2^ are red-shifted. Moreover, the A_g_^2^ phonon mode becomes much broader after SiN layer deposition. The change in the Raman spectra is mainly due to the doping effect and possible compressive strain introduced into the SnSe flake during capping layer deposition. A similar shift in the B_3g_ and A_g_^2^ phonon modes was observed by pressure-dependent Raman spectroscopy.^27^

**Fig. 3:**
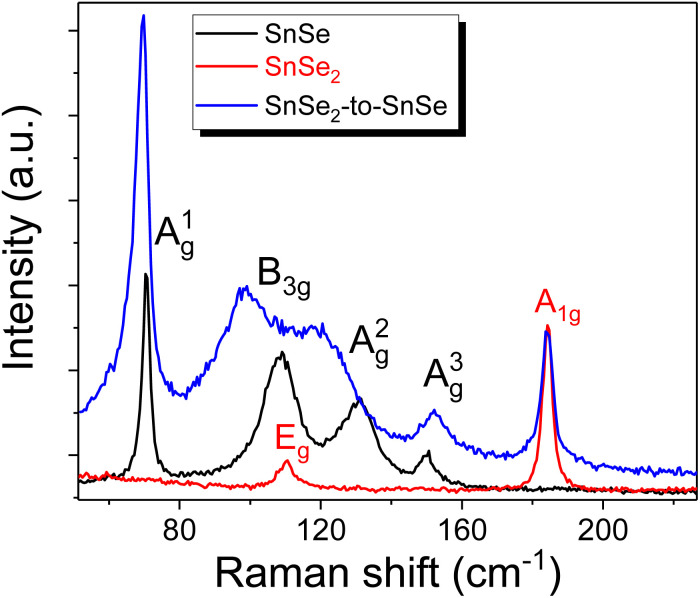
Micro-Raman spectra taken from as-exfoliated SnSe and SnSe_2_ flakes, and from SnSe_2_ flakes after NH_3_ plasma treatment at 300 °C for 10 s with a plasma power of 20 W and a partial pressure of 700 mTorr.

The unit cell of SnSe_2_ contains one Sn atom and two Se atoms, which means that nine vibrational modes are expected; two acoustic phonon modes, four optical modes of which A_1g_ + E_g_ are Raman-active, and two more A_2u_ + E_u_ infrared-active modes. The Raman-active modes are the in-plane E_g_ at 118 cm^−1^, indicating that our SnSe_2_ is composed of a 1T phase, and the out-of-plane phonon mode A_1g_ at 185 cm^−1^ (see Fig. 2a and b). The SiN_*x*_ capping layer has no influence on the phonon structure of SnSe_2_. Fig. 3 exemplarily shows the micro-Raman spectra obtained from the SnSe_2_ flake after NH_3_ plasma treatment for 10 s at 300 °C with a plasma power of 20 W. The gas pressures in the chamber were fixed at 700 mTorr. For comparison, the Raman spectra obtained from unprocessed SnSe and SnSe_2_ are shown. In the case of the unprocessed samples, the Raman spectra show the well-known Raman-active phonon modes in both SnSe and SnSe_2_, such as the one presented in Fig. 2a. However, the Raman spectrum obtained from the SnSe_2_ flake after NH_3_ plasma treatment exhibits five well-resolved phonon modes located at 69.7, 98.7, 121.3, 152.2, and 184.2 cm^−1^. The first four phonon modes have a similar position to the A_g_^1^, B_3g_, A_g_^2^ and A_g_^3^ phonon modes in SnSe, while the last phonon mode at 184.2 cm^−1^ overlaps with the Raman-active A_1g_ phonon mode in SnSe_2_. The E_g_ phonon mode from SnSe_2_ is not well visible in the plasma-treated sample due to the overlapping with the B_3g_ and A_g_^2^ phonon modes from SnSe. The coexistence of the SnSe- and SnSe_2_-related phonon modes in the Raman spectrum suggest that the top layer of the trigonal SnSe_2_ flake was converted to an orthorhombic SnSe layer during the NH_3_-plasma treatment. Moreover, the red shift of the main phonon modes of SnSe and the slight broadening of the A_g_^1^ peak (the FWHM increases from 2.9 cm^−1^ to 4.6 cm^−1^ after plasma treatment) indicate tensile strain and defect formation in the top layer, *e.g.* Se vacancies that are created during the conversion from SnSe_2_ to SnSe. The lattice parameters of trigonal SnSe_2_ are *a* = *b* = 3.81 Å, and *c* = 6.14 Å, while the lattice parameters of orthorhombic SnSe are *a* = 11.5 Å, *b* = 4.15 Å, and *c* = 4.44 Å.^28^ Using DFT calculations, Tian *et al.* showed that the SnSe_2_–SnSe heterojunction possesses an atomically flat interface with a well-defined rotation angle between the layers influencing the lattice mismatch and strain accumulation in the converted layer.^29^ The NH_3_ plasma can be decomposed into different positively charged ions including NH_*n*_^+^_(0≤*n*≤3)_, H_2_^+^, H^+^ and N^+^ during processing. To clarify the influence of the different plasma components on the phase transformation process in chalcogenides, the influence of nitrogen plasma on the stability of the SnSe and SnSe_2_ flakes was also inspected.

The N_2_-plasma parameters were the same as those used for the NH_3_ plasma. According to Raman spectroscopy, the N_2_-plasma treatment of SnSe_2_ even for 2 min does not affect the microstructural properties of the flakes (not shown here). This indicates that ionized hydrogen is primarily responsible for the transformation of Sn-dichalcogenide into Sn-monochalcogenide through the formation of volatile hydrogen-chalcogenide molecules. Kim *et al.* investigated the phase transformation in SnS_2_ flakes applying an Ar plasma.^22^ The phase transformation of SnS_2_ was observed to occur for a plasma power larger than 60 W. It was also shown that phase transformation depends on plasma power and time. For a plasma power of about 140 W, the SnS phase appears after a few seconds, but the plasma treatment with 60 W needs at least 120 s. However, the Ar-plasma-treated surface turned out to be rough, showing a columnar growth of SnS on top of SnS_2_, which is due to the selective sputtering of the 2D material and not because of the chemical reaction as in our case. A similar phenomenon was observed in works related to the SnS_2_ heat treatment *via* laser irradiation. Laser heating causes selective S evaporation and the phase transformation from SnS_2_ into SnS.^30,31^ Using an NH_3_ plasma we can precisely control the thickness of the converted layer and the interface between the dichalcogenide and the monochalcogenide is almost atomically sharp, as shown later by cross-sectional TEM. The NH_3_ gas dissociates in the plasma chamber into NH_*n*≤3_^+^, H_2_^+^, H^+^ and N^+^ ions. Hydrogen ions are highly mobile and can easily diffuse into SnSe_2_. Hydrogen reacts with Se atoms, reducing SnSe_2_ to SnSe. The released Se–H_*x*_ at an elevated temperature diffuses to the surface and tends to form stable but volatile H_2_Se molecules that are pumped out from the chamber.

**Fig. 4:**
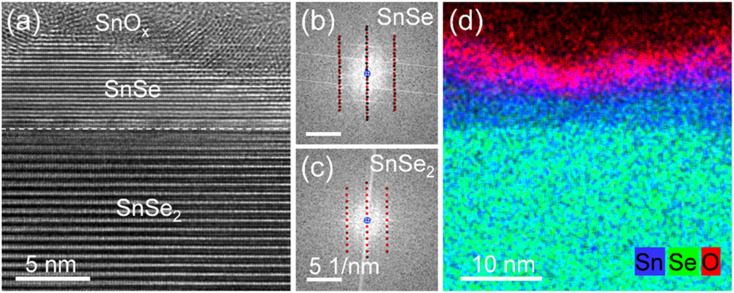
(a) Cross-sectional high-resolution TEM image of a SnSe_2_ flake after NH_3_-plasma treatment at 300 °C for 10 s. (b) and (c) fast Fourier transforms obtained from the SnSe and SnSe_2_ sample regions indicated in panel (a) and superimposed with simulated diffraction patterns of orthorhombic SnSe and trigonal SnSe_2_ in [031] and [210] zone axis geometry, respectively. (d) Superimposed EDX-based element distributions of Sn (blue), Se (green), and oxygen (red).

Fig. 4a shows a cross-sectional high-resolution TEM image of the vertical p–n junction formed by NH_3_-plasma-induced phase transformation of SnSe_2_. In particular, the sample was treated with an NH_3_ plasma at 300 °C for 10 s. After this plasma process, about 5–10 nm of the top layer of SnSe_2_ is converted to SnSe with an atomically flat interface between the two materials. Crystalline SnO_*x*_ is formed on the top of the SnSe layer due to surface oxidation (Fig. 4d). Fig. 4b and c show fast Fourier transforms (FFTs) obtained from the converted and virgin SnSe_2_ sample regions depicted in Fig. 4a. In particular, the FFTs are described by [031] and [210] zone axis patterns of SnSe and SnSe_2_, respectively, confirming the successful phase transformation from trigonal dichalcogenide into orthorhombic monochalcogenide by NH_3_ plasma treatment. Furthermore, from the element distribution from EDX results (as shown in Fig. 4d), it could be found that the amounts of the Se element at the surface were much lower compared with that of Se in the inner layer. This also indicates the successful phase transformation of SnSe_2_.

**Fig. 5:**
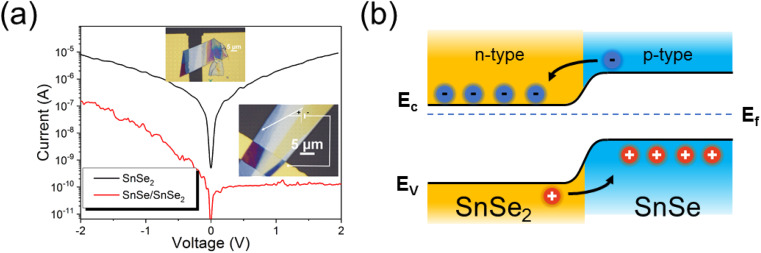
(a) *I*–*V* characteristics of an as-exfoliated SnSe_2_ flake (black curve) and a sample after NH_3_-plasma treatment. The upper inset shows the SnSe_2_ flake on gold electrodes, and the bottom inset shows the flake after NH_3_-plasma treatment with a schematic electrical connection. One electrode is connected to the gold pads and the second one with a needle connects the part of the SnSe_2_ flake fully transformed into SnSe. (b) Schematic diagram of the band gap alignment for the SnSe/SnSe_2_ p–n junction.

Next, we performed an electrical characterization of the SnSe_2_ flakes before and after NH_3_-plasma treatment. For this experiment, SnSe_2_ was exfoliated and subsequently transferred onto a pre-patterned substrate with Au contacts (see upper inset of Fig. 5a). Fig. 5a shows the current–voltage (*I*–*V*) curves obtained from an as-deposited SnSe_2_ flake (black curve) and from a flake after NH_3_-plasma treatment (red curve). As-grown SnSe_2_ is an n-type semiconductor with a Fermi level of about 0.02 eV below the conduction band minimum, while the non-intentionally-doped SnSe shows p-type conductivity with a Fermi level of about 0.1 eV above the valence band maximum indicating heavy electron and hole doping, respectively.^18,29^ It was shown that in both cases Se vacancies are mainly responsible for the doping. Therefore, after the successful transformation of a part of SnSe_2_ into SnSe, the formation of an n–p junction is expected.

The current–voltage measurement for the untreated SnSe_2_ layer shows a typical characteristic for unipolar semiconductors (black curve). The red curve shows the *I*–*V* characteristic of the p–n junction made of SnSe/SnSe_2_. In this case, the thick SnSe_2_ flake was transferred onto one Au-contact pad only, so that the thicker part of the flake was bonded to the Au-stripe and the thinner part was lying on SiN_*x*_. The *I*–*V* measurements were performed using a probe station with a microscope in which one probe tip came into contact with the Au-stripe (to connect with SnSe_2_), while the second probe tip came into contact with p-type SnSe at the end of the flake (see the bottom inset of Fig. 5). Micro-Raman spectroscopy also shows that the blue part of the flake visible in the lower inset of Fig. 5, where the probe tip is touching the flake, contains only SnSe. The *I*–*V* curve obtained from the NH_3_-plasma-treated sample shows typical p–n junction characteristics with a rectification factor in the order of 10^3^ and an ideality factor *n* = 6.5. An ideality factor above 2 is commonly observed for the 2D heterojunctions due to the interface states and the electrical contact quality between metals and 2D material.^32^ Optimization of the contact quality was not the focus of the current manuscript. Fig. 5b shows the energy diagram of the SnSe/SnSe_2_ p–n junction. Taking into account the band offset and the position of the Fermi levels in both semiconductors, the SnSe/SnSe_2_ p–n junction forms a type-II band alignment.^22^ The presented *I*–*V* curves clearly demonstrate the successful phase transformation of SnSe_2_ into SnSe and the formation of a p–n junction.

## Conclusions

We have demonstrated that n-type SnSe_2_ flakes can be converted to p-type SnSe flakes using a few seconds of NH_3_-plasma treatment. The process is precisely controllable and reproducible and leads to an atomically flat SnSe_2_/SnSe interface. The formed vertical p–n junction shows a rectification factor in the order of 10^3^. The developed method can be used to fabricate both lateral and vertical p–n junctions just by using local masking or adjusting the plasma parameters. The process can be easily scaled up and used to fabricate devices at the wafer scale with different functionalities.

## Conflicts of interest

There are no conflicts to declare.

## Supplementary Material
